# Single-center experience with immune checkpoint inhibitor-related ureteritis and cystitis

**DOI:** 10.3389/fimmu.2025.1727822

**Published:** 2026-01-20

**Authors:** Chenyu Wang, Muwen Nie, Yuan Liu, Wei Qiu, Zhiyang Zhang, Na Zhou, Xiang Wang, Lin Zhao, Hongyan Ying, Chunmei Bai

**Affiliations:** 1 Department of Medical Oncology, Peking Union Medical College Hospital, Chinese Academy of Medical Sciences & Peking Union Medical College, Beijing, China; 2 Department of Internal Medicine, Peking Union Medical College Hospital, Chinese Academy of Medical Sciences & Peking Union Medical College, Beijing, China

**Keywords:** corticosteroid, ICI-related ureteritis and cystitis, immune check point inhibitors, immune-related adverse event, JAK inhibitor

## Abstract

**Backgroud:**

Ureteritis and cystitis is a rare immune-related adverse event (irAE) of immune checkpoint inhibitors (ICIs), challenging to distinguish from urinary tract infection (UTI), easily leading to missed diagnosis. We aim to describe clinical features, radiological characteristics and treatment of patients who suffer from ICI-related ureteritis and cystitis (ICI-UC).

**Methods:**

This was a single centre case series of patients diagnosed with solid tumor who received ICIs treatment and subsequently suffered from ICI-UC. All clinical demographic data, laboratory parameters, imaging characteristics, and treatment information were collected.

**Results:**

Between Mar 1^st^, 2020 and Mar 31^th^ 2025, 12 of 1239 patients treated at Peking Union Medical College Hospital with ICIs were confirmed to have ICI-related ureteritis and cystitis (0.96%), 10 males and 2 females. Only 1 patient received anti-programmed cell death protein 1 (PD-1) and cytotoxic T-lymphocyte associated protein-4 (CTLA-4) dual immunotherapy, the other 11 patients received PD-1/PD-ligand 1 plus chemotherapy or/and target therapy. The median time to onset was 83 days (range 28–442 days). All patients (100%) exhibited significant urinary tract irritation symptoms. 12 patients demonstrated characteristic imaging abnormalities, including hydroureteronephrosis, irregular ureteral wall thickening, bladder wall thickening with irregular margins, or/and conspicuous renal fascia. Spontaneous remission was observed in 2 patients, while 10 patients received steroids and all showed rapid improvement of symptom after treatment.The median time from symptom onset to the initiation of steroids was 24 days, and the median prednisone dose was 0.60 mg/kg/day. Six patients (6/10, 60%) experienced disease recurrence during the corticosteroid tapering phase, and two patients who failed steroid tapering were successfully treated with a combination of corticosteroid and JAK inhibitor therapy.

**Conclusion:**

This pioneering cohort study provides the first systematic investigation of ICI-UC, establishing its incidence and comprehensively characterizing clinical and imaging features. Through cohort analysis, we propose a novel severity grading system with corresponding treatment algorithms, while additionally exploring the therapeutic potential of JAK inhibitors for steroid-dependent cases.

## Introduction

Immune checkpoint inhibitors (ICIs), a novel advancement in cancer therapy, activate T cell-mediated antitumor immune responses by blocking immune checkpoints. such as cytotoxic T-lymphocyte-associated protein 4 (CTLA-4), programmed cell death protein 1 (PD-1)/programmed death-ligand 1 (PD-L1), leading to a marked improvement in clinical outcomes across multiple advanced cancers (1). However, this nonspecific immune activation may lead to immune system attacks on healthy tissues, triggering a spectrum of immune-related adverse events (irAEs). Skin, gastrointestinal tract and endocrine organs are commonly affected (2, 3).

With the expanding clinical use of ICIs, urinary tract involvement has emerged as a newly recognized phenotype of rare irAE, which was first reported in 2017 (4). Early reports characterized this irAE as isolated cystitis, but subsequent evidence demonstrated this irAE could involved the entire urothelium (5, 6). This irAE has been variably termed as (nonbacterial) cystitis, uretitis, ureteritis/cystitis, or cystoureteritis in previous case reports (7–9). For consistency in this study, we will uniformly refer to this condition as immune checkpoint inhibitor related uretitis and cystitis (ICI-UC). Currently, fewer than 30 cases of ICI-UC have been reported in the literature. The majority of reported ICI-UC cases were associated with anti-PD-1 monotherapy, with additional cases reported following anti-PD-L1 therapy or PD-1/CTLA-4 blockade (10–14). The cycles prior to symptom onset varied widely from 1 to 77 (12, 15).

The diagnosis of ICI-UC remains clinically challenging, patients usually presented with symptoms such as dysuria, urinary frequency, hematuria, suprapubic or lower lumbar pain, often mimicking bacterial cystitis. However, urinalysis cannot reliably differentiate between these two conditions. Although cystoscopy and biopsy were performed in some cases, findings were non-specific (4, 5, 12). Key distinctions included sterile urine cultures and history of ICI administration.

Common irAEs already have standard grading systems, such as checkpoint inhibitor pneumonitis (CIP), however, current literature uniformly employed Common Terminology Criteria for Adverse Events (CTCAE) for ICI-UC severity assessment relying on symptoms (such as urinary frequency, hematuria) (14, 16). The lack of consensus grading system for ICI-UC contributed to significant heterogeneity in therapeutic decision-making. While corticosteroids remained the mainstay of ICI-UC management, significant variations existed in initial dosing and treatment duration (17–19). Furthermore, clinical experience remains limited for steroid-dependent cases, posing unique therapeutic challenges (15).

While emerging case reports provided recognition of ICI-UC, though systematic characterization remains lacking. This is the first single-center cohort focus on ICI-UC, we aim to characterize the onset and clinical features of ICI-UC, explore distinctive imaging characteristics and establish the grading system and therapeutic framework for this emerging toxicity.

## Materials and methods

This single-institution case series of adult solid tumor patients treated at the Department of Oncology, Peking Union Medical College Hospital (PUMCH), Chinese Academy of Medical Sciences (CAMS). The study protocol received approval from the PUMCH Institutional Review Board(I-25PJ1701).

**Figure 1:**
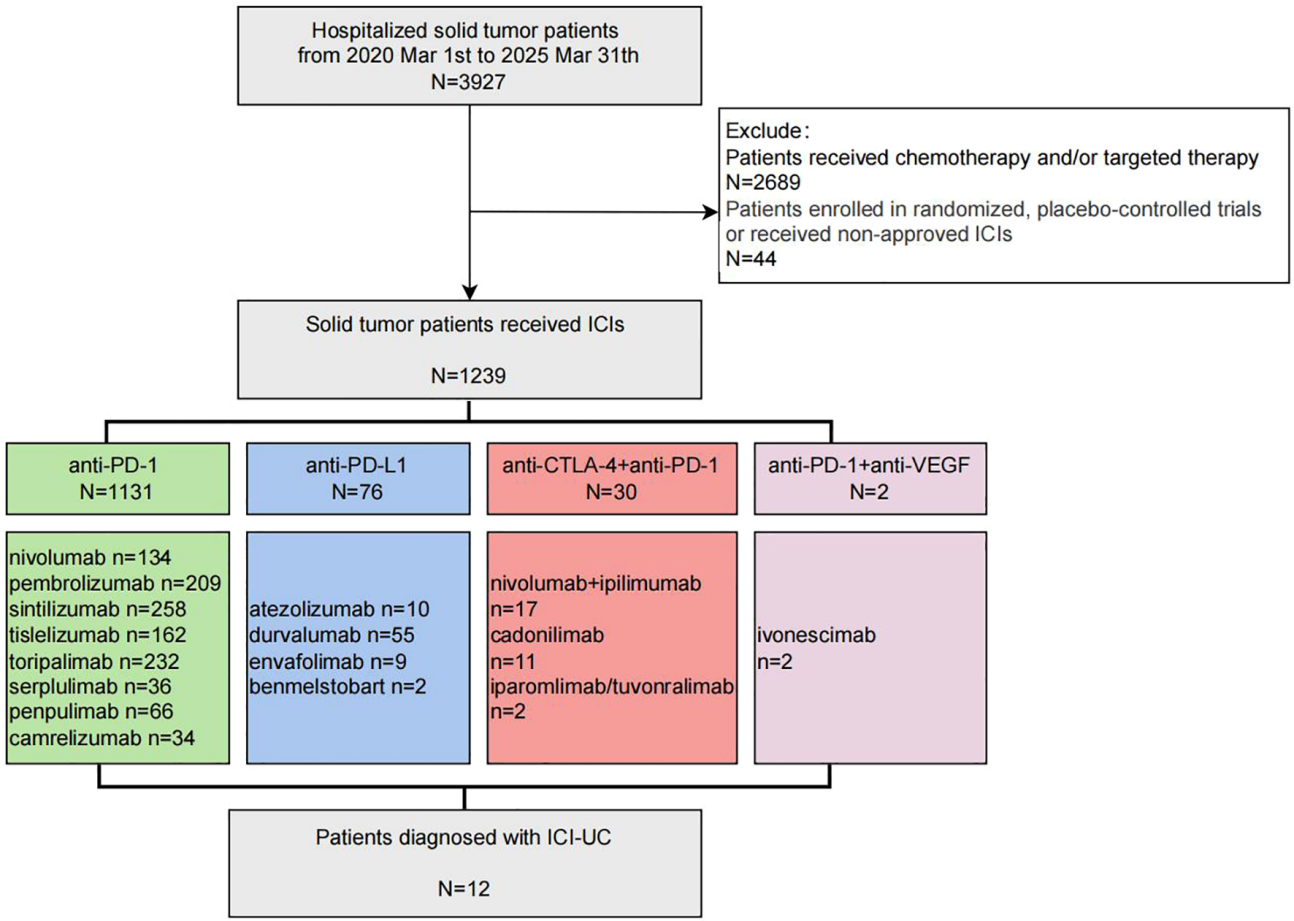
Flowchart of patients enrollment.

We identified patients treated with ICIs through a review of electronic medical records between Mar 1st, 2020 to Mar 31th, 2025, the flowchart was shown in **Figure 1**. The ICIs included PD-1 monoclonal antibody (sintilimab, pembrolizumab, nivolumab, tislelizumab, toripalimab, serplulimab, camrelizumab, and penpulimab), PD-L1 monoclonal antibody (atezolizumab, durvalumab, envafolimab and benmelstobart), CTLA-4 antibody (ipilimumab), PD-1/CTLA-4 bispecific antibody (cadonilimab, iparomlimab/tuvonralimab) and PD-1/VEGF bispecific antibody (ivonescimab), patients enrolled in randomized controlled trials or receiving non-approved immunotherapies were excluded from the analysis.

After screening ICI-treated cohort, we identified ICI-UC patients through the following criteria: 1) prominent urinary tract irritation symptoms after receiving ICI treatment; 2) dilation of the renal pelvis and ureters and/or the ureteral or bladder wall appeared thickened; 3) negative urine culture and ineffective antibiotics treatment. Patients were excluded due to the following reasons: 1) Presence of underlying urological conditions that could cause similar symptoms, such as symptomatic benign prostatic hyperplasia, urinary tract stones, neurogenic bladder, or urinary tract infection (UTI); 2) History of radiation-induced bladder injury; 3) History of invasive urological procedures; 4) Imaging or cystoscopy suggested a primary tumor or structural changes in the urinary tract caused by tumor; 5) Immunotherapy discontinuation for at least 3 months; 6) History of systemic NSAID or immunosuppressant or medications affecting lower urinary tract function (e.g., alpha-blockers, anticholinergics) use within 3 months before ICI-UC symptom oneset.

For the included patients, information about demographic characteristics, disease stage, ICI type, combined treatment, treatment response, combined irAEs, analysis of urine, peak value of creatinine, radiology presentations including computed tomography (CT) and/or ultrasound findings of the urinary system, cystoscopic appearance, treatment of ICI-UC, overall survival (OS) were collected. Staging was based on the American Joint Committee on Cancer’s Cancer Staging Manual, 8th edition. The onset time of ICI-UC was calculated from ICI initiation to the beginning symptom of ICI-UC. OS was defined as the time from diagnosis to death from any cause.

## Results

### Patients demographics and oncologic outcomes

From Mar 1st, 2020 to Mar 31th, 2025, 1239 patients had received ICIs (**Supplementary Table 1**), A total of 12 patients (10 male and 2 female) were diagnosed with ICI-UC, accounting for 0.96% of the ICI-treated population. As **Table 1** shown, the median age was 61.5 (range 30-78). None of these patients had past medical history of urinary tract diseases. Malignancies included 5 gastric cancers (all proficient mismatch repair (pMMR), including 3 HER2-positive patients), 1 head and neck cancer, 2 pancreatic cancer, 1 small cell lung cancer, 1 cholangiocarcinoma and 1 microsatellite instability-high (MSI-H) small intestinal cancer, all the patients were diagnosed with stage IV at initiation of ICI treatment. 10 of 12 patients received anti-PD-1 monoclonal antibody (3 nivolumab, 3 sintilimab, 2 penpulimab, 1 toriparimab and 1 pembrolizumab) combined with chemotherapy, 1 patient received anti-PDL1 monoclonal antibody (durvalumab) combined with etoposide and cisplatin regimen and 1 patient received PD-1/CTLA-4 dual-checkpoint blockade (nivolumab and ipilimumab). Except the patient who received PD-1/CTLA-4 dual-checkpoint blockade, all other 11 patients received combination of ICI and chemotherapy (7 patients nab-paclitaxel based regimen, 58.3%), besides, 3 HER2-positive gastric cancer patients (25.0%) received Trustuzumab and 2 pancreatic cancer patient (16.7%) received anlotinib simutaneously in a single-arm phase II study. In terms of best overall response, complete remission (CR) was observed in 1 patient, partial remission (PR) in 5 patients, stable disease (SD) in 5 patients, and progressive disease (PD) in 1 patient. At the cutoff date of May 31th, 2025, with a median follow-up of 46.5 months, median OS was 51.8m (95%CI 25.1m ~ 78.5m) for the all 12 patients.

**Table 1:** Baseline clinical charcteristics of patients prior to development of ICI-UC.

No.	Disease	Stage	ICIs	Combined treatment	Line	Cycles prior to onset	Days prior to onset	Combined irAEs	Best efficacy	Survival (months)
1	Gastric cancer	Stage IV	Sintilimab	Nab-paclitaxel+S-1	2nd	2	28	G1 hypothyroidism	PR	Alive (19.6)
2	Laryngeal cancer	Stage IV	Pembrolizumab	Nab-paclitaxel+carboplatin	1st	4	109	G1 hypothyroidism G2 Myositis	PR	Alive (55.3)
3	Pancreatic cancer	Stage IV	Penpulimab	Nab-paclitaxel+anlotinib	4th	2	42	G1 Hyperthyroidism	PD	Dead (51.8)
4	HER2-positive gastric cancer	Stage IV	Sintilimab	Trustuzumab+XELOX	1st	2	29	–	SD	Dead (18.9)
5	HER2-positive gastric cancer	Stage IV	Nivolumab	Trustuzumab+nab-paclitaxel	2nd	6	165	G1 Hypothyroidism G1 Rash	CR	Alive (48.2)
6	Small cell lung cancer	Stage IV	Durvalumab	EP	1st	5	103	–	PR	Dead (33.9)
7	Gastric cancer	Stage IV	Nivolumab	SOX	1st	4	104	–	SD	Dead (15.8)
8	Small intestinal cancer	Stage IV	Nivolumab+ Ipilimumab	–	2nd	3	49	–	PR	Alive (5.6)
9	Gastric cancer	Stage IV	Nivolumab	Nab-paclitaxel	2nd	4	63	–	SD	Alive (18.1)
10	Pancreatic cancer	Stage IV	Penpulimab	Nab-paclitaxel+anlotinib	4th	9	250	G1 myocarditis G1 hypothyroidism	SD	Alive (36.7)
11	HER2-positive gastric cancer	Stage IV	Sintilimab	Trustuzumab+nab-paclitaxel+S-1	2nd	14	442	–	SD	Dead (25.2)
12	Cholangiocarcinoma	StageIV	Toripalimab	Gemcitabine+capetabine	1st	1	21	–	PR	Alive (45.6)

ICI, immune checkpoint inhibitor; XELOX, oxaliplatin and capetabine; EP, etoposide and cisplatin; SOX, oxaliplatin and tegafur/gimercil/oteracil potassium; S-1, tegafur/gimercil/oteracil potassium; Line, The line of anti-tumor therapy; Cycles, The number of ICI cycles; Days prior to onset, The time from the first ICI dose to symptom onset; irAEs, Immune-Related Adverse Event.

### Onset and clinical features of ICI-UC

The median time to onset of ICI-UC was 83 days (range 28–442 days), median number of cycles prior to onset of ICI-UC was 4 (range 2-14) (**Table 1**). 12 patients (100%) presented with prominent urinary tract irritation symptoms such as urinary frequency, urgency, dysuria or difficulty urinating. 4 patients (33.3%) manifested with notable gross hematuria. 4 patients (33.3%) experienced with low back pain. The diagnosis of ICI-UC was complicated, all of 12 patients showed negative urine culture and had received at least one empirical antibiotic treatment, with 4 patients (25.0%) having been treated with three different antibiotics. Considering urinalysis, all of 12 patients demonstrated microscopic hematuria, proteinuria and leukocyturia. Among 12 patients, 6 underwent 24-hour urine protein (24hUP) quantification, median 2.48g, span from 0.82g to 3.75g. 8 patients (8/12, 66.7%) experienced creatine elevated (median 1.6mg/dL, range 1.2-2.6mg/dL) (**Table 2**).

**Table 2:** Serological, imaging and cystoscopic features of ICI-UC.

No.	Creatine (umol/L)	Urine WBC (Cells/ul)	Urine RBC (Cells/ul)	Urine Pro (g/L)	24hUP (g)	Features of CT scan	PVR (ml)	Cystoscopy	ANA	Other antibody	Urine cytology
1	Elevated (119)	1+	3+	2+	1.45	①②③④	18	Mucosa course	Negative	ANCA (–)	Negative
2	Elevated (154)	3+	3+	2+	NA	①②③④	5	Multiple lymphoid follicles	C 1:80	PCNA(+) ANCA/GBM (-)	NA
3	Normal (44)	1+	3+	3+	NA	①③	22.5	NA	Negative	ANCA(-)	Negative
4	Elevated (170)	3+	3+	3+	NA	①②③④	NA	Mucosa course hyperemia	S 1:160	ANCA/GBM (-)	NA
5	Normal (97)	1+	2+	1+	NA	①②③④	NA	NA	S 1:80	ANCA(-)	NA
6	Elevated (233)	2+	3+	2+	0.82	①②③④	NA	hyperemia	S 1:80	ANCA/GBM/PLA2R(-)	Negative
7	Elevated (115)	3+	3+	2+	3.75	①②④	5	rough/hyperemia	Negative	ANCA(-)	Negative
8	Elevated (126)	3+	1+	2+	NA	①②③④	NA	NA	Negative	ANCA(-)	Negative
9	Normal (81)	3+	3+	3+	2.05	①②③④	16	NA	Negative	ANCA(-) BP180(-)	NA
10	Normal	3+	3+	1+	NA	①②③	NA	hyperemia/ edema	Negative	ANCA(-)	NA
11	Elevated (208)	1+	3+	2+	2.90	①②③④	NA	hyperemia	Negative	ANCA/GBM (-)	NA
12	Elevated (107)	2+	3+	2+	3.41	①③	21.7	NA	NA	NA	NA

**Figure 2:**
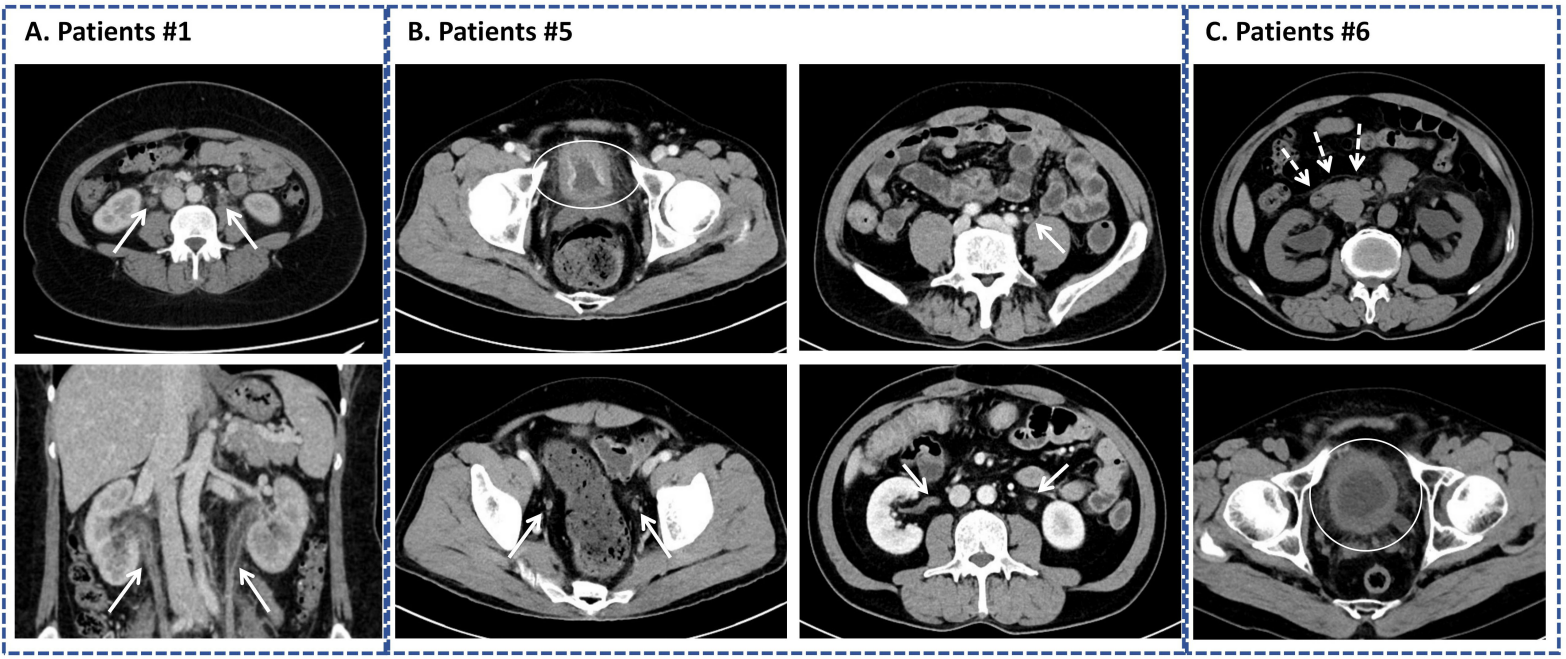
Characteristic computed tomography (CT) manifestation of immune checkpoint inhibitors (ICIs)-related ureteritis and cystitis. **(A)** Patients No. 1. **(B)** Patient No.5 with mild urinary irritative symptoms. **(C)** Patient No.6 with visualization of the anterior renal fascia.Thickening of bladder wall is outlined by a white oval. (⇢) represents anterior renal fascia. (→) represents thickening or enhancement of the ureteral wall and dilation of the renal pelvis and ureter.

① Dilation of the renal pelvis and ureter; ② Thickening or enhancement of the ureteral wall;③ Thickening of bladder wall;④ Visualization of the anterior renal fascia;

24hUP, 24-hour urinary protein; ANA, antinuclear antibody;ANCA, antineutrophil cytoplasmic antibody;GBM, glomerular basement membrane;PLA2R, hospholipase A2 receptor; PVR, Post-Void Residual;

All patients underwent ultrasound and non-contrast or contrast-enhanced CT to evaluate urinary tract. Computed tomography (CT) findings of ICI-UC include the following features: 1) dilation of the renal pelvis and ureters, ureters exhibited “beaded appearance”, typically involving bilateral sides; 2) the ureteral walls appeared thickened with irregular margins, often demonstrating abnormal enhancement on contrast-enhanced CT; 3) The bladder wall often appears thickened and irregular, with poor distension; 4) the renal fascia can be visualized on CT imaging (**Table 2**), featured CT manifestation was shown in **Figure 2**. The presentation of cystoscopy including hyperemia (5 patients), mucosa rough (3 patients), edema (1 patient) and multiple lymphoid follicles (1 patient). Residual urine was measured by ultrasound in 6 out of 12 patients, with a median postvoid residual (PVR) volume of 17 mL (range 5-22.5ml). The combination of negative urine cultures and ≥2 characteristic CT findings demonstrated 100% diagnostic specificity in our cohort.

Among 11 patients, 4 (36.4%) were ANA-positive at baseline, with low-level positivity, range from 1:80 to 1:160. Patient 2 tested positive for anti-proliferating cell nuclear antigen (PCNA) antibodies, no positivity detected for anti-neutrophil cytoplasmic antibodies (ANCA) (11 patients), anti-Glomerular Basement Membrane (GBM) (4 patients), anti-phospholipase A2 receptor (PLA2R) (1 patient) or anti-bullous pemphigoid 180 (BP180) (1 patient) (**Table 2**).

### Combined other irAEs

5 of 12 patients (41.7%) developed other immune-related adverse events (irAEs) concurrently or sequentially during immunotherapy, in addition to cystitis and ureteritis. 5 patients presented with thyroid immune-related adverse events (Grade 1), 1 patient experienced with myositis (Grade 2), and 1 patient developed myocarditis (Grade 1) with only cardiac troponin I increased (as **Table 1** shown).

### Treatment of ICI-UC

**Figure 3:**
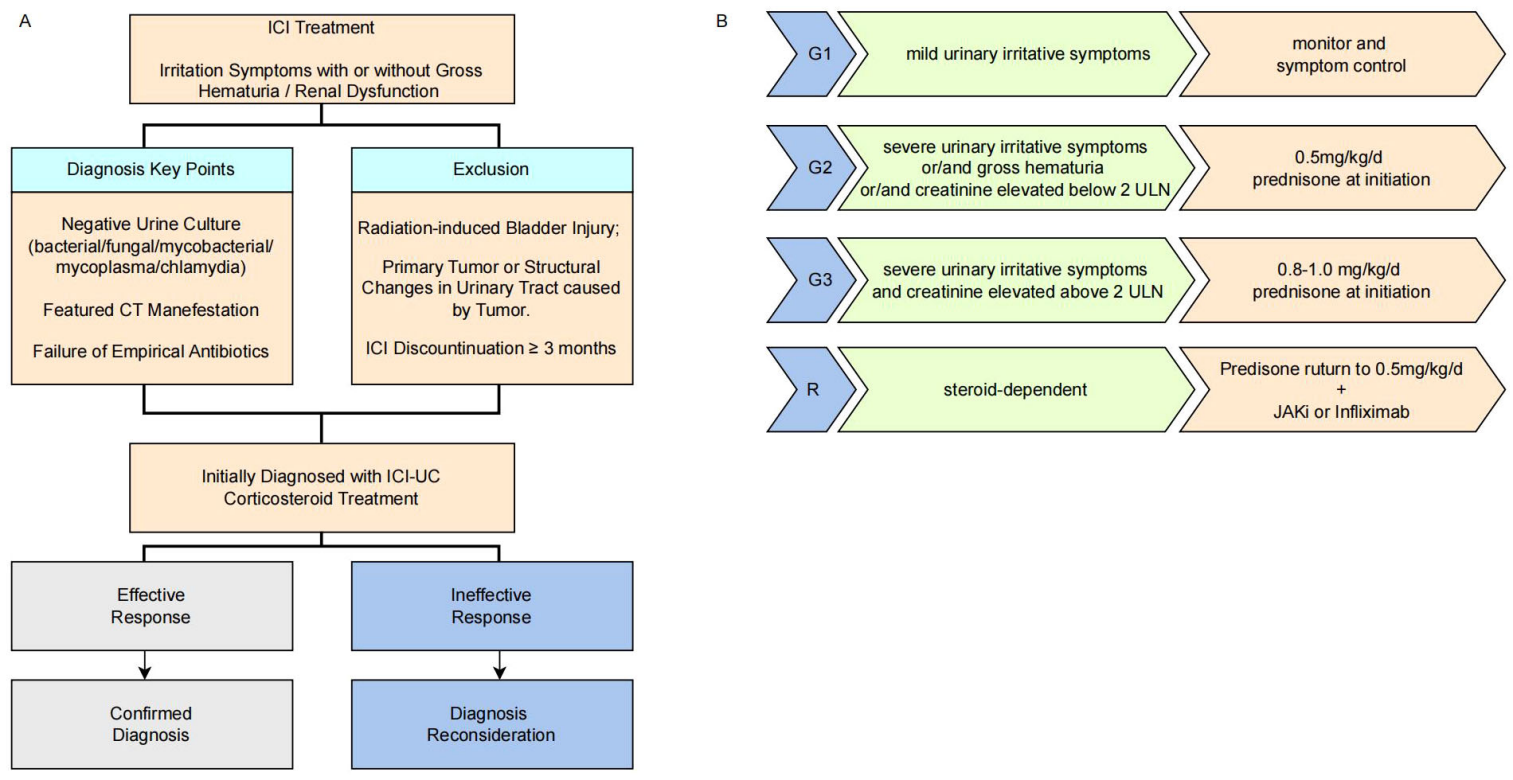
Diagnostic workflow for ICI-UC **(A)**, grading and therapeutic strategies for ICI-UC **(B)**.

As **Table 3** shown, Two patients (No. 2 and No. 5) demonstrated a spontaneous resolution of symptoms without corticosteroid treatment at onset, presenting with mild urinary frequency and pain. Clinically, they exhibited classic urinalysis and CT findings indicative of ICI-UC, CT imaging was shown in **Figure 2A**. Both cases resolved within approximately one month, allowing them to continue ICI treatment without symptom recurrence. Out of 12 patients, 10 (83.3%) had received steroids treatment. The median time from symptom onset to the initiation of steroids was 24 days, and the median prednisone dose among these patients was 0.60 mg/kg/day (ranging from 0.15 to 0.96 mg/kg/day). Six patients experienced disease recurrence during the corticosteroid tapering phase, necessitating dose re-escalation. Among which, 2 patients received salvage therapy with prednisone re-escalation and Janus kinase (JAK) inhibitors (tofacitinib 5mg twice a day) simultaneously. Patient No. 9 experienced a disease flare-up as prednisone was tapered to 5 mg/day. Given the planned subsequent surgical intervention, the patient was transitioned to a low-dose steroid and JAK inhibitor regimen (prednisone 15 mg/day and tofacitinib 5 mg twice daily) instead of a high-dose steroid re-challenge. The patient maintained disease remission with rapid steroid discontinuation and JAK inhibitor maintenance, successfully underwent surgery without experiencing an ICI-UC flare, based on our experience we set up a grading system and relative treatment instruction as **Figure 3** shown.

**Table 3:** Treatment modalities for ICI-UC.

No.	Time from onset to steroid (days)	Number of antibiotics type	D-J stent	ICI continued/ discontinued	Treatment	Symptom resolved (time, days)	Recurrent	Treatment after recurrent
1	50	3	Yes	Discontinued	PSL 30mg/d (80kg, 0.375mg/kg), tapered gradually to 10mg in 8 weeks and relapsed	Yes (7 days)	Yes	PSL 60mg/d JAKi
2	–	1	No	Continued	Spontaneous resolution without PSL	Yes	No	–
3	15	2	No	Discontinued	PSL 10mg/d (70kg, 0.15mg/kg/d)×2 weeks and discontinued after symptoms relieved	Yes (14 days)	Yes	PSL 20mg/d for 2weeks
4	131	3	No	Discontinued	PSL 30mg/d (55kg, 0.55mg/kg/d), tapered gradually to 10mg in 8 weeks and reduced by 2.5mg qd every 2 weeks	Yes (3 days)	No	–
5	–	1	No	Continued	Spontaneous resolution without PSL	Yes	No	–
6	14	1	No	Discontinued	PSL 60mg/d (78.5kg, 0.76mg/kg/d)×10 d tapered gradually in 6 weeks	Yes (3 days)	Yes	PSL 60mg/d
7	12	1	No	Discontinued	PSL 50mg/d (69kg, 0.72mg/kg/d)×10 d tapered gradually to 25mg/d in 6 weeks	Yes (3 days)	Yes	PSL 50mg/d
8	17	2	No	Discontinued	MP 60mg/d (85kg, 0.88mg/kg/d)×10 d tapered gradually to 5mg/d in 6 weeks	Yes (10 days)	Yes	PSL 15mg/d+ JAKi
9	5	2	No	Discontinued	PSL 30mg/d (57kg, 0.53mg/kg/d) No obvious decrease in 24UP, PSL increased to 55mg/d (0.96/kg/d), gradully tapered in 8 weeks	Yes (1 day)	No	–
10	31	3	No	Discontinued	PSL 45mg/d (69kg, 0.65mg/kg/d), tapered gradully in 6 weeks	Yes	No	–
11	48	1	Yes	Discontinued	PSL 30mg/d (60kg, 0.5mg/kg/d), tapered gradully in 10 weeks	Yes (Symptom relief after D-J removed)	No	–
12	44	3	Yes	Continued	PSL 30mg/d (63kg, 0.48mg/kg/d), corticosteroid tapering regimen not available	Yes	Yes	D-J stent & persistent symptoms

ICI, immune checkpoint inhibitor; PSL, prednisone; MP: Methylprednisolone; D-J stent, Double-J stent; JAKi, Janus kinase inhibitor.

## Discussion

Currently, due to the low incidence rate and insufficient awareness among physicians, ICI-UC were only reported as individual cases. To our knowledge, this is the largest single-center cohort of ICI-UC, encompassing various aspects such as incidence, time of onset, clinical manifestations, imaging features, treatment and oncological outcomes.

Currently, the most widely used immune checkpoint inhibitors in clinical practice include PD-1/PD-L1 inhibitors and CTLA-4 inhibitors. However, patients may experience irAEs during ICI therapy, and irAEs could affect any organ system (20). The administration of PD-1/PD-L1 inhibitors was associated with an overall irAE incidence rate of 66.0% for all-grade events and 14.0% for grade ≥3 adverse events, and the incidence of irAEs of all grade and grade ≥3 adverse events for PD-1/PD-L1 plus CTLA-4 combination therapy was relatively higher with 89% and 67% (21, 22), the most commonly affected organs in immunotherapy include the skin, thyroid and colon (23). Some rare irAEs have also been documented, including type 1 diabetes mellitus (0.48%) and renal irAEs (around 1%) (24). However, only case reports of ICI-UC were available previously, the incidence and clinical characteristics of ICI-UC could not be fully understood, through our cohort, the incidence was first reported 0.95%. Due to limited number of ICI-UC patients, the study did not calculate the incidence separately in CTLA-4 plus PD-1 subgroup and PD-1/PD-L1 inhibitor subgroups. The majority of irAEs occur within 1–3 months after treatment initiation, early-onset irAEs (median time to onset approximately 1 month) include myocarditis and myositis, while delayed-onset events such as pancreatitis and diabetes mellitus typically manifest later, with a median onset of approximately 4 months (25). The median time to onset of ICI-UC was 83 days, but the onset exhibited a wide temporal distribution (range 28–442 days). Among 28 ICI-UC patients identified from 24 case reports, the median time to symptom onset was 5.5 cycles (range: 1–77 cycles), in our cohort, the onset cycle was 4 (4–19, 22, 26–33). The earliest-onset previous case report was a cholangiocarcinoma patient who developed urinary irritation symptoms just 8 days after initiating toripalimab (a PD-1 monoclonal antibody) plus ipilimumab combination therapy (12).

Considering the similarities between UTI and ICI-UC, the diagnosis of ICI-UC was highly challenging, with a median time of 24 days from symptom onset to the initiation of empirical glucocorticoid therapy in our study. All ICI-UC cases clinically presented with urinary irritation symptoms and exhibited varying degrees of urinary laboratory abnormalities, which was indistinguishable from urinary tract infection (UTI), but no patients had fever in our study. Majority of patients (66.7%) presented with mild to moderate elevation in serum creatinine levels (median 1.6mg/dL, range 1.2-2.6mg/dL), commonly demonstrating ureteral and pelvic dilation, which indicated postrenal obstruction could be considered as a potential cause of creatinine elevation. Considering creatine elevation, it is necessary to differentiate it from ICI-related acute kidney injury (ICI-AKI). ICI-AKI typically lacks irritative urinary symptoms, with less common microscopic hematuria and higher peak serum creatinine level (median 4.5 mg/dL, IQR [3.6–7.3 mg/dl]) (34). Meanwhile, cystoscopic findings and inconclusive biopsy pathology were non-specific. Based on our experience, the most diagnostically significant features for ICI-UC were repeated negative urine cultures and CT findings. ICI-UC exhibited highly characteristic imaging manifestations such as bladder and/or ureteral wall thickening with irregular margins, involvement of the anterior renal fascia may be observed when cases with severe inflammation, which were described in previous cases (10, 30). Urine culture and microbiological workup typically require extended processing time, thus metagenomic next-generation sequencing was also used to exclude UTI in a case report, which could facilitate rapid clinical judgment and exhibited promising apllied significance (10). Besides, failure of empirical antibiotic therapy and rapid symptomatic relief with corticosteroid were key distinctive features to confirm the diagnosis of ICI-UC (7, 35).

Drug-induced hemorrhagic cystitis most commonly occurs with cyclophosphamide and ifosfamide, but the cystitis induced by taxane-based treatment was rare (36, 37). Prior case reports have documented albumin-bound paclitaxel-induced cystitis with gross hematuria, however, onset of cystitis induced by nab-paclitaxel was early (3–10 days), urine leukocyte was negative and low dose of prednisone demonstrated clinical efficacy with no featured ultrasound or CT manifestation (38, 39). In our cohort, 7 out of 12 patients (58.3%) had received nab-paclitaxel based regimens. And several patients in previous case reports about ICI-UC received ICIs combined with paclitaxel-based chemotherapy (5, 7, 10, 14, 19, 32). Whether ICIs and taxane-based chemotherapy play a synergistic role in the development of ICI-UC remains unclear. In addition, attention should be paid to patients with a history of pelvic radiotherapy, to differentiate from radiation-induced cystitis (40).

The potential mechanisms of immune checkpoint inhibitor (ICI)-induced cystitis and ureteritis, as inferred from existing case reports and literature reviews, primarily center on the dysregulation of immune tolerance and aberrant immune activation triggered by ICI therapy. ICIs disrupt the PD-1/PD-L1 signaling pathway to enhance antitumor immunity, but this process concurrently breaks down self-tolerance, leading to autoimmune attacks on normal urothelial and ureteral tissues (10, 14, 20). Key pathological findings, such as predominant infiltration of CD3^-^ and CD8^-^ cytotoxic T lymphocytes (and occasionally TIA-1^-^ lymphocytes) in the bladder and ureteral mucosa, support the role of activated T cells in mediating tissue damage by recognizing unknown autoantigens expressed on urinary tract epithelial cells (8, 19, 26). Emerging evidence suggested certain autoantibodies may correlate with the development of immune-related adverse events after the application of ICIs (41). Patients with positive antithyroid antibodies showed higher immune-related thyroid adverse effect rates (75.0% vs 13.8%). Tahir et al. demonstrated serological associations of anti-CD74 with pneumonitis (42). A significant elevation in anti-BP180 antibodies and anti-integrin α6β4 antibodies levels, which was associated with tight junction structure attaching urothelial basal cells to the basement membrane, was observed in a case of immune checkpoint inhibitor (ICI)-induced ureteritis and cystitis (17). However, in our study, a patient had negative result of BP180, which underscore the need to identify potential biomarkers of ICI-UC to facilitate differential diagnosis.

Current management of ICI-UC remains primarily based on case reports (**Table 4**), highlighting an urgent need for systematic studies to establish standardized grading criteria and treatment guidelines. However, CTCAE criteria fails to comprehensively reflect the full spectrum and clinical severity of irAEs (43). Based on our cohort and experience, we have preliminarily established a grading system and therapeutic strategies for ICI-UC, illustrated as **Figure 3**. Mild urinary tract irritation symptoms may potentially resolve spontaneously without ICI discountinuation (30). For patients with urinary tract irritation symptoms affecting quality of life, gross hematuria, or elevated creatinine, corticosteroid therapy may be required as initial treatment. Based on current reported cases of ICI-UC, patients generally achieve rapid symptomatic relief following corticosteroid therapy (5, 9, 10, 14, 15, 17, 35). However, disease recurrence during steroid tapering has been observed, majority of patients attempted steroid rechallenge. Based on this phenomenon, despite rapid initial response to corticosteroids, a gradual 6–8 week taper is mandatory to prevent rebound, even in clinically improved patients. However, prolonged corticosteroid therapy may potentially compromise the efficacy of immunotherapy, biological agents may represent a viable therapeutic alternative, a steroid-dependent ICI-UC patient successfully managed through combination therapy incorporating infliximab (15). Tofacitinib is a JAK-STAT inhibitor and has shown efficacy in immune-related adverse events, which demonstrated extraordinary clinical remission rate in steroid-resistant patients (96.7%) and patients with steroid taper failure (100%) (44). The two ICI-UC cases with steroid taper failure in our cohort showed great clinical remission with Tofacitinib and successful steroid taper.

**Table 4:** Clinical features and treatment of case reports from previous literatures.

No.	Author	Disease	Stage	ICIs	Combined treatment	Cycles prior to onset	Treatment	symptom resolution time (days)	ICI	Relapse	Ref
1	X He	Lung SCC	IIIB	Pembrolizumab	CCRT	6	MP 40mg qd*3	NA	Rechallenge	Yes	(29)
2	Keisuke Ozak	Lung SCC	IV	Nivolumab	No	3	PSL 0.5mg/kg, taper gradully	NA	Continued	No	(4)
3	Fan Y	ICC	IV	Toripalimab Ipilimumab	No	1	MP 120 mg/d×5d→80 mg/d×3 d, graudully taper	5 days	Discontinued	Yes	(12)
4	Obayashi A	TNBC	IV	Atezolizumab	Nab-paclitaxel	4	PSL 40mg/d×4d→30mg/d×7d, symptom recur→60mg/d	2 days	Discontinued	No	(14)
5	Zhu S	ICC	IV	Atezolizumab	Lenvatinib	3	2mg/kg/d, taper slowly	NA	Discontinued	No	(13)
6	Li J	ESCC	IV	Tislelizumab	Doxetaxel+ nedaplatin	6	MP 60mg/d×2w and taper gradully	3 days	Discontinued	Yes	(10)
7	Li J	GC	IV	Sintilimab	Paclitaxel+S-1	3	MP 60mg/d×7d and taper gradully	7 days	Discontinued	Yes	
8	Li J	GC	IV	Nivolumab	No	2	MP 60mg/d×7d	3 days	Discontinued	Yes	
9	Zhang P	Lung SCC	IV	Sintilimab	Paclitaxel	6	MP 40mg/d×3d and gradully reduced in 8 weeks	4 days	Discontinued	No	(17)
10	Zhang P	Lung SCC	III	Pembrolizumab	Nab-paclitaxel	6	MP 40mg/d×2d and gradully reduced in 8 weeks	4 days	Rechallenge	Yes	
11	Shimatani K	Lung SCC	IV	Nivolumab	No	7	PSL 60mg/d	NA	Continued But failed	Yes	(6)
12	Shimatani K	Lung SCC	IV	Nivolumab	No	12	Stop and relief	NA	Discontinued	No	(6)
13	Zhu L	SCLC	IV	Nivolumab	Paclitaxel	5	MP 80mg twice a day ×3d and gradully reduced in 6 weeks	3 days	Discontinued	No	(19)
14	Schneider S	melanoma	–	Nivolumab Ipilimumab	No	2	Discontinued PSL (0.5 mg/kg/d)	NA	Rechallenge	NA	(11)
15	Fukunaka H	Lung cancer	IIIB	Nivolumab	No	77	60 mg/day (1 mg/kg)	immediately	Discontinued	Yes	(45)
16	Anraku T	salivary duct carcino-ma	IVB	Pembrolizumab	No	2	spontaneous resolution After bladder hydrodistension	NA	Discountined	No	(26)
17	Ji J	GC	III	Sintilimab	Oxaplatin+S-1	3	spontaneous resolution	NA	Discountined	No	(30)
18	Yajima S	Lung adenocarcinoma	IV	Nivolumab	No	18	symptoms disappeared after biopsy	NA	Continued	No	(33)
19	Ueki Y	Lung adenocarcinoma	IV	Pembrolizumab	No	17	PSL 25mg/d, taper 2months	19 days	Continued	No	(8)
20	Tu L	Lung adenocarcinoma	IV	Sintilimab	Nab-paclitaxel+Bevacuzumab	3	MP 80mg/d, taper in 8weeks	2 days	Discontinued	No	(5)
21	Wang Z	GC	IV	Sintilimab	Nab-paclitaxel+S-1	5	Chinese medicine Chai-Ling-Tang	NA	Continued	No	(32)
22	Di C	Lung adenocarcinoma	IVB	Pembroalizumb	Pemetrexed + carboplatin	5	Observation and showed relief after discoutinued	NA	Continued	Yes	(27)
23	Zhou Q	Thymic cancer	IV	Tislelizumab	Nab-paclitaxel+ carboplatin	6	MP 1mg/kg/d×3d and reduced to PSL 12mg, tapered gradully in 40 days	3 days	Discontinued	No	(7)
24	Kihira H	HER2- Positive GC	IV	Nivolumab	Oxaplatin+S-1	6	MP 50 mg(1 mg/kg/day)	quickly	Discontinued	No	(35)
25	Alhusari L	Lung adenocarcinoma	IIB	Pembrolizumb	Pemetrexed + carboplatin	8	PSL 1mg/kg/d and tapered off	NA	Discontinued	No	(18)
26	Ni C	Pancreatic cancer	IV	Sintilimab	Gemcitabine	6	Dexamethasone 5mg/d×6d	6 days	Rechallenge	No	(31)
27	El Husseini K	NSCLC	IV	Pembrolizumb	No	5	PSL 1 mg/kg/day	NA	Discontinued	No	(28)
28	Li S	Lung adenocarcinoma	IVA	Pembrolizumb	Pemetrexed + carboplatin	14	PSL 30mg/d×14d and reduced by 5mg per week	2 days	NA	NA	(9)

CRT, concurrent chemoradiotherapy; ESCC, esophageal squamous cell cancer; GC: gastric cancer;PSL, prednisone; MP, Methylpredniso- lone; ICC, intrahepatic cholangiocarcinoma; ICI, immune checkpoint inhibitor; NSCLC, non-small cell lung cancer; SCC, squamous cell cancer; TNBC, triple-negative breast cancer; S-1, tegafur/gimercil/oteracil potassium.

### Limitation

This study has the following limitations: 1) As a single-center retrospective analysis, it provided preliminary insights into the incidence and clinical characteristics of ICI-UC. However, due to limited understanding of this condition, it is possible some ICI-UC patients were not included in our study, and treatment approaches were highly individualized. Therefore, we plan to conduct a prospect study to optimize therapeutic strategies. 2) In patients with ICI-UC who exhibited mild-to-moderate creatinine elevation, we primarily attribute this to postrenal obstruction secondary to ureteral/bladder wall thickening. Notably, these renal function abnormalities demonstrated rapid resolution following corticosteroid therapy. However, the potential coexistence of ICI-associated acute kidney injury (ICI-AKI) in this setting requires exclusion through confirmatory renal biopsy. 3) Our study lacked histopathological characterization of bladder or ureteral mucosal biopsies because the procedure at our institution requires general anesthesia and rigid cystoscopy. To minimize patient risk, we prioritized clinical diagnosis based on: medication history, characteristic clinical presentation, diagnostic imaging features, steroid responsiveness, and this approach maintained diagnostic accuracy while reducing invasive procedures.

## Conclusion

This article represents the first cohort study specifically investigating ICI-UC, we report incidence rate for the first time and make a detailed description of ICI-UC clinical and imaging characteristics. Based on our cohort analysis and existing literature, we have established a grading system and treatment strategy for ICI-UC, besides, we give a insight in therapeutic potential of JAK inhibitors in steroid-dependent ICI-UC.

## Data Availability

The raw data supporting the conclusions of this article will be made available by the authors, without undue reservation.

## Supplementary material

Supplementary Table 1 baseline characteristics of the ICI-treated patients in this study Other cancers include Thymic Carcinoma;Adrenocortical Carcinoma; Neuroendocrine Neoplasm;Soft Tissue Sarcoma;Cutaneous Squamous Cell Carcinoma;Ovarian Cancer;Mesothelioma;Thyroid Cancer;Melanomal Cervical Cancer

## References

[1] Sullivan RJ Weber JS . Immune-related toxicities of checkpoint inhibitors: mechanisms and mitigation strategies. Nat Rev Drug Discov. (2022) 21:495–508. doi: 10.1038/s41573-021-00259-5, PMID: 34316029

[2] Geisler AN Phillips GS Barrios DM Wu J Leung DYM Moy AP . Immune checkpoint inhibitor-related dermatologic adverse events. J Am Acad Dermatol. (2020) 83:1255–68. doi: 10.1016/j.jaad.2020.03.132, PMID: 32454097 PMC7572894

[3] Alruwaii ZI Montgomery EA . Gastrointestinal and hepatobiliary immune-related adverse events: A histopathologic review. Adv Anat Pathol. (2023) 30:230–40. doi: 10.1097/PAP.0000000000000401, PMID: 37037419

[4] Ozaki K Takahashi H Murakami Y Kiyoku H Kanayama H . A case of cystitis after administration of nivolumab. Int Cancer Conf J. (2017) 6:164–6. doi: 10.1007/s13691-017-0298-6, PMID: 31149494 PMC6498356

[5] Tu L Ye Y Tang X Liang Z You Q Zhou J . Case report: A case of sintilimab-induced cystitis/ureteritis and review of sintilimab-related adverse events. Front Oncol. (2021) 11:757069. doi: 10.3389/fonc.2021.757069, PMID: 35004277 PMC8733470

[6] Shimatani K Yoshimoto T Doi Y Sonoda T Yamamoto S Kanematsu A . Two cases of nonbacterial cystitis associated with nivolumab, the anti-programmed-death-receptor-1 inhibitor. Urol Case Rep. (2018) 17:97–9. doi: 10.1016/j.eucr.2017.12.006, PMID: 29541592 PMC5849865

[7] Zhou Q Qin Z Yan P Wang Q Qu J Chen Y . Immune-related adverse events with severe pain and ureteral expansion as the main manifestations: a case report of tislelizumab-induced ureteritis/cystitis and review of the literature. Front Immunol. (2023) 14:1226993. doi: 10.3389/fimmu.2023.1226993, PMID: 37869004 PMC10587548

[8] Ueki Y Matsuki M Kubo T Morita R Hirohashi Y Sato S . Non-bacterial cystitis with increased expression of programmed death-ligand 1 in the urothelium: An unusual immune-related adverse event during treatment with pembrolizumab for lung adenocarcinoma. IJU Case Rep. (2020) 3:266–9. doi: 10.1002/iju5.12211, PMID: 33163921 PMC7609190

[9] Li S Zheng K Xu Y Wang M . Immune checkpoint inhibitors related cystoureteritis: A case report and literature review. Zhongguo Fei Ai Za Zhi. (2023) 26:709–16. doi: 10.3779/j.issn.1009-3419.2023.106.17, PMID: 37985157 PMC10600747

[10] Li J Yu YF Qi XW Du Y Li CQ . Immune-related ureteritis and cystitis induced by immune checkpoint inhibitors: Case report and literature review. Front Immunol. (2022) 13:1051577. doi: 10.3389/fimmu.2022.1051577, PMID: 36685488 PMC9853439

[11] Schneider S Alezra E Yacoub M Ducharme O Gerard E Dutriaux C . Aseptic cystitis induced by nivolumab and ipilimumab combination for metastatic melanoma. Melanoma Res. (2021) 31:487–9. doi: 10.1097/CMR.0000000000000765, PMID: 34433197

[12] Fan Y Zhao J Mi Y Zhang Z Geng Y Zhou L . Recurrent cystitis associated with 2 programmed death 1 inhibitors: A rare case report and literature review. J Immunother. (2023) 46:341–5. doi: 10.1097/CJI.0000000000000484, PMID: 37721343 PMC10540753

[13] Zhu S Bian L Lv J Liu B Shen J . A case report of non-bacterial cystitis caused by immune checkpoint inhibitors. Front Immunol. (2021) 12:788629. doi: 10.3389/fimmu.2021.788629, PMID: 35003107 PMC8733335

[14] Obayashi A Hamada-Nishimoto M Fujimoto Y Yoshimoto Y Takahara S . Non-bacterial cystitis with increased expression of programmed cell death ligand 1 in the urothelium: an unusual immune-related adverse event after atezolizumab administration for metastatic breast cancer. Cureus. (2022) 14:e25486. doi: 10.7759/cureus.25486, PMID: 35800819 PMC9246443

[15] Fukunaga H Sumii K Kawamura S Okuno M Taguchi I Kawabata G . A case of steroid-resistant cystitis as an immune-related adverse event during treatment with nivolumab for lung cancer, which was successfully treated with infliximab. IJU Case Rep. (2022) 5:521–3. doi: 10.1002/iju5.12532, PMID: 36341187 PMC9626353

[16] Li Y Jia X Zhang Y Du Y Chang Y Shen Y . Risk factors and immunomodulators use in steroid-refractory checkpoint inhibitor pneumonitis. J Immunother Cancer. (2023) 11(6):e006982. doi: 10.1136/jitc-2023-006982, PMID: 37290926 PMC10254972

[17] Zhang P Yin C Yang M . Case reports of immune-related cystitis and the antibody combination hypothesis. Immunotherapy. (2024) 16:1039–47. doi: 10.1080/1750743X.2024.2389761, PMID: 39263930 PMC11492643

[18] Alhusari L Abdallah M Nwanwene K Shenouda M . Acute non-infectious cystitis secondary to immune-related adverse events in a patient receiving pembrolizumab for treatment of non-small cell lung cancer: A case report. Cureus. (2024) 16:e55666. doi: 10.7759/cureus.55666, PMID: 38586668 PMC10997305

[19] Zhu L Wang Z Stebbing J Wang Z Peng L . Immunotherapy-related cystitis: case report and review of the literature. Onco Targets Ther. (2021) 14:4321–8. doi: 10.2147/OTT.S321965, PMID: 34366676 PMC8336986

[20] Postow MA Sidlow R Hellmann MD . Immune-related adverse events associated with immune checkpoint blockade. N Engl J Med. (2018) 378:158–68. doi: 10.1056/NEJMra1703481, PMID: 29320654

[21] Mi Z Zhang Y Feng Z Liu J Wu J Tan H . Treatment-related adverse events of PD-1/PD-L1 inhibitors combined with CTLA-4 inhibitors in clinical trials: a meta-analysis. Artif Cells Nanomed Biotechnol. (2022) 50:301–9. doi: 10.1080/21691401.2022.2131354, PMID: 36217590

[22] Yin Q Wu L Han L Zheng X Tong R Li L . Immune-related adverse events of immune checkpoint inhibitors: a review. Front Immunol. (2023) 14:1167975. doi: 10.3389/fimmu.2023.1167975, PMID: 37304306 PMC10247998

[23] Suijkerbuijk KPM van Eijs MJM van Wijk F Eggermont AMM . Clinical and translational attributes of immune-related adverse events. Nat Cancer. (2024) 5:557–71. doi: 10.1038/s43018-024-00730-3, PMID: 38360861

[24] Kamitani F Nishioka Y Koizumi M Nakajima H Kurematsu Y Okada S . Immune checkpoint inhibitor-related type 1 diabetes incidence, risk, and survival association. J Diabetes Investig. (2025) 16:334–42. doi: 10.1111/jdi.14362, PMID: 39569589 PMC11786175

[25] Gougis P Jochum F Abbar B Dumas E Bihan K Lebrun-Vignes B . Clinical spectrum and evolution of immune-checkpoint inhibitors toxicities over a decade-a worldwide perspective. EClinicalMedicine. (2024) 70:102536. doi: 10.1016/j.eclinm.2024.102536, PMID: 38560659 PMC10981010

[26] Anraku T Hashidate H Imai T Kawakami Y . Successful treatment of immune-related cystitis with bladder hydrodistension. IJU Case Rep. (2023) 6:211–5. doi: 10.1002/iju5.12588, PMID: 37405028 PMC10315250

[27] Di C Yu T Ni L . Non-bacterial cystitis caused by pembrolizumab therapy for adenocarcinoma of the lung: a case report. Front Immunol. (2024) 15:1423123. doi: 10.3389/fimmu.2024.1423123, PMID: 39034999 PMC11257856

[28] El Husseini K Lafoeste H Mansuet-Lupo A Arrondeau J Villeminey C Bennani S . A case of severe interstitial cystitis associated with pembrolizumab. In: Curr Probl Cancer Case Rep. (2021) 4:100101.

[29] He X Tu R Zeng S He Z Liu S Fang Y . Non-bacterial cystitis secondary to pembrolizumab: A case report and review of the literature. Curr Probl Cancer. (2022) 46:100863. doi: 10.1016/j.currproblcancer.2022.100863, PMID: 35687965

[30] Ji J Lai CH Zhang X Hu H . Immune-related adverse events with renal colic as the main manifestation: a case report of sintilimab-induced ureteritis/cystitis treated by ureteral stent and review of the literature. Front Immunol. (2024) 15:1501415. doi: 10.3389/fimmu.2024.1501415, PMID: 39763683 PMC11700999

[31] Ni CX Zhao Y Qian H Fu H Yan YY Qiu YS . Long survival in a pancreatic carcinoma patient with multi-organ toxicities after sintilimab treatment: A case report. Front Pharmacol. (2023) 14:1121122. doi: 10.3389/fphar.2023.1121122, PMID: 36744247 PMC9894891

[32] Wang Z Zhu L Huang Y Peng L . Successful treatment of immune-related cystitis by Chai-Ling-Tang (Sairei-To) in a gastric carcinoma patient: Case report and literature review. Explore (NY). (2023) 19:458–62. doi: 10.1016/j.explore.2022.04.002, PMID: 35469747

[33] Yajima S Nakanishi Y Matsumoto S Tanabe K Sugano M Masuda H . Improvement of urinary symptoms after bladder biopsy: A case of pathologically proven allergy-related cystitis during administration of nivolumab. IJU Case Rep. (2021) 4:213–5. doi: 10.1002/iju5.12286, PMID: 34308272 PMC8294138

[34] Murakami N Motwani S Riella LV . Renal complications of immune checkpoint blockade. Curr Probl Cancer. (2017) 41:100–10. doi: 10.1016/j.currproblcancer.2016.12.004, PMID: 28189263 PMC5440194

[35] Kihara H Yoshino S Kitahara M Sakamoto K Nagashima Y Yahara N . A case of advanced gastric cancer who experienced multiple immune-related adverse events with nivolumab and SOX therapy. Gan To Kagaku Ryoho. (2023) 50:1804–6., PMID: 38303213

[36] Traxer O Desgrandchamps F Sebe P Haab F Le Duc A Gattegno B . Hemorrhagic cystitis: etiology and treatment. Prog Urol. (2001) 11:591–601. 11761677

[37] Ntekim AI Ajekigbe A . Hemorrhagic cystitis in a patient receiving docetaxel for prostate cancer. Clin Med Insights Oncol. (2010) 4:11–3. doi: 10.4137/CMO.S4477, PMID: 20567631 PMC2883243

[38] Ichioka E Iguchi-Manaka A Oikawa T Sawa A Okazaki M Saito T . A case of hemorrhagic cystitis caused by nab-paclitaxel. Int Cancer Conf J. (2016) 5:187–91. doi: 10.1007/s13691-016-0255-9, PMID: 31149452 PMC6498339

[39] Zhang XJ Lou J . Hemorrhagic cystitis in gastric cancer after nanoparticle albumin-bound paclitaxel: A case report. World J Gastrointest Oncol. (2024) 16:1084–90. doi: 10.4251/wjgo.v16.i3.1084, PMID: 38577472 PMC10989392

[40] Pascoe C Duncan C Lamb BW Davis NF Lynch TH Murphy DG . Current management of radiation cystitis: a review and practical guide to clinical management. BJU Int. (2019) 123:585–94. doi: 10.1111/bju.14516, PMID: 30113758

[41] Les I Martínez M Pérez-Francisco I Cabero M Teijeira L Arrazubi V . Predictive biomarkers for checkpoint inhibitor immune-related adverse events. Cancers (Basel). (2023) 15(5):1629. doi: 10.3390/cancers15051629, PMID: 36900420 PMC10000735

[42] Tahir SA Gao J Miura Y Blando J Tidwell RSS Zhao H . Autoimmune antibodies correlate with immune checkpoint therapy-induced toxicities. Proc Natl Acad Sci U S A. (2019) 116:22246–51. doi: 10.1073/pnas.1908079116, PMID: 31611368 PMC6825284

[43] Hsiehchen D Watters MK Lu R Xie Y Gerber DE . Variation in the assessment of immune-related adverse event occurrence, grade, and timing in patients receiving immune checkpoint inhibitors. JAMA Netw Open. (2019) 2:e1911519. doi: 10.1001/jamanetworkopen.2019.11519, PMID: 31532516 PMC6751757

[44] Verheijden RJ van Eijs MJM May AM van Wijk F Suijkerbuijk KPM . Immunosuppression for immune-related adverse events during checkpoint inhibition: an intricate balance. NPJ Precis Oncol. (2023) 7:41. doi: 10.1038/s41698-023-00380-1, PMID: 37173424 PMC10182067

[45] Yuasa T . Editorial Comment to A case of steroid-resistant cystitis as an immune-related adverse event during treatment with nivolumab for lung cancer, which was successfully treated with infliximab. IJU Case Rep. (2022) 5:524. doi: 10.1002/iju5.12538, PMID: 36341185 PMC9626351

